# Radiomics-based risk prediction: survival in sarcopenic gastrointestinal and genitourinary cancer cohorts using a population-specific sarcopenia definition

**DOI:** 10.1186/s41747-026-00848-2

**Published:** 2026-11-13

**Authors:** Nishta Letchumanan, Shouhei Hanaoka, Kotaro Sugawara, Satoru Taguchi, Wataru Gonoi, Mariko Kurokawa, Shohei Inui, Shinichiro Shiomi, Koichi Yagi, Yoshifumi Baba, Kenjiro Kishitani, Haruki Kume, Takeharu Yoshikawa, Osamu Abe

**Affiliations:** 1 Department of Radiology, Graduate School of Medicine, The University of Tokyo, Tokyo, Japan; 2 Department of Radiology, The University of Tokyo Hospital, Tokyo, Japan; 3 Department of Gastrointestinal Surgery, Graduate School of Medicine, The University of Tokyo, Tokyo, Japan; 4 Department of Urology, Graduate School of Medicine, The University of Tokyo, Tokyo, Japan; 5 Department of Computational Diagnostic Radiology and Preventive Medicine, The University of Tokyo Hospital, Tokyo, Japan

**Keywords:** Kaplan–Meier estimate, Radiomics, Risk assessment, Sarcopenia, Survival analysis

## Abstract

**Objective:**

This study demonstrated that the recently proposed Asian (Japanese)-cohort-based skeletal muscle index (SMI) definition (< 36.2 ${{\rm{cm}}}^{2}\rm/{m}^{2}$ for males and < 29.6 ${{\rm{cm}}}^{2}\rm/{m}^{2}$ for females), along with other factors such as radiomic and clinical variables, could improve risk categorization for survival in sarcopenic, gastrointestinal (GI), and genitourinary (GU) cancer patients.

**Materials and methods:**

This retrospective study involved 2,015 patients who underwent major surgery at our institution between 2006 and 2018. From the axial CT image of the L3 vertebral region, the skeletal muscle was segmented to calculate the SMI values. 646 radiomic features were extracted from the image, and the Boruta algorithm was used to identify the most pertinent features. The final set of features consisted of these features and clinical variables. The dataset was split into training and testing sets comprising 1,512 patients (75%) and 503 patients (25%), respectively. Four models were evaluated in this study: (1) random survival forest (RSF)-based radiomic model, (2) Cox proportional hazard-based model, (3) Asian (Japanese)-specific SMI definition-based model, and (4) integrated model, which combines experiments (2) and (3). The Kaplan–Meier (KM) curves, log-rank tests, and Harrell’s concordance index (C-index) were computed to assess the predictive performance of the models.

**Results:**

The integrated model achieved the highest performance in terms of C-index (0.760, 95% CI: 0.686–0.835) among all other models. Survival stratification was statistically significant across all the experiments (*p* < 0.0001).

**Conclusion:**

This work demonstrates the efficacy of utilizing recently proposed Japanese population-based SMI definition values to optimize risk stratification for sarcopenic patients.

**Key Points:**

***Question*** Can population-specific sarcopenia definitions, clinical variables, and CT muscle radiomics together improve overall survival risk stratification in patients with gastrointestinal and genitourinary cancers?

***Findings*** In this cohort of 2,015 patients, combining sarcopenia, clinical variables, and CT muscle radiomics yielded an overall survival C-index of 0.760.

***Relevance statement*** The incorporation of the Asian (Japanese)-specific skeletal muscle index definition improves risk stratification for the survival of sarcopenic cancer patients when combined with an imaging-based approach, such as radiomics and other clinical parameters, highlighting the value of multimodal integration.

**Graphical Abstract:**

**Figure:**
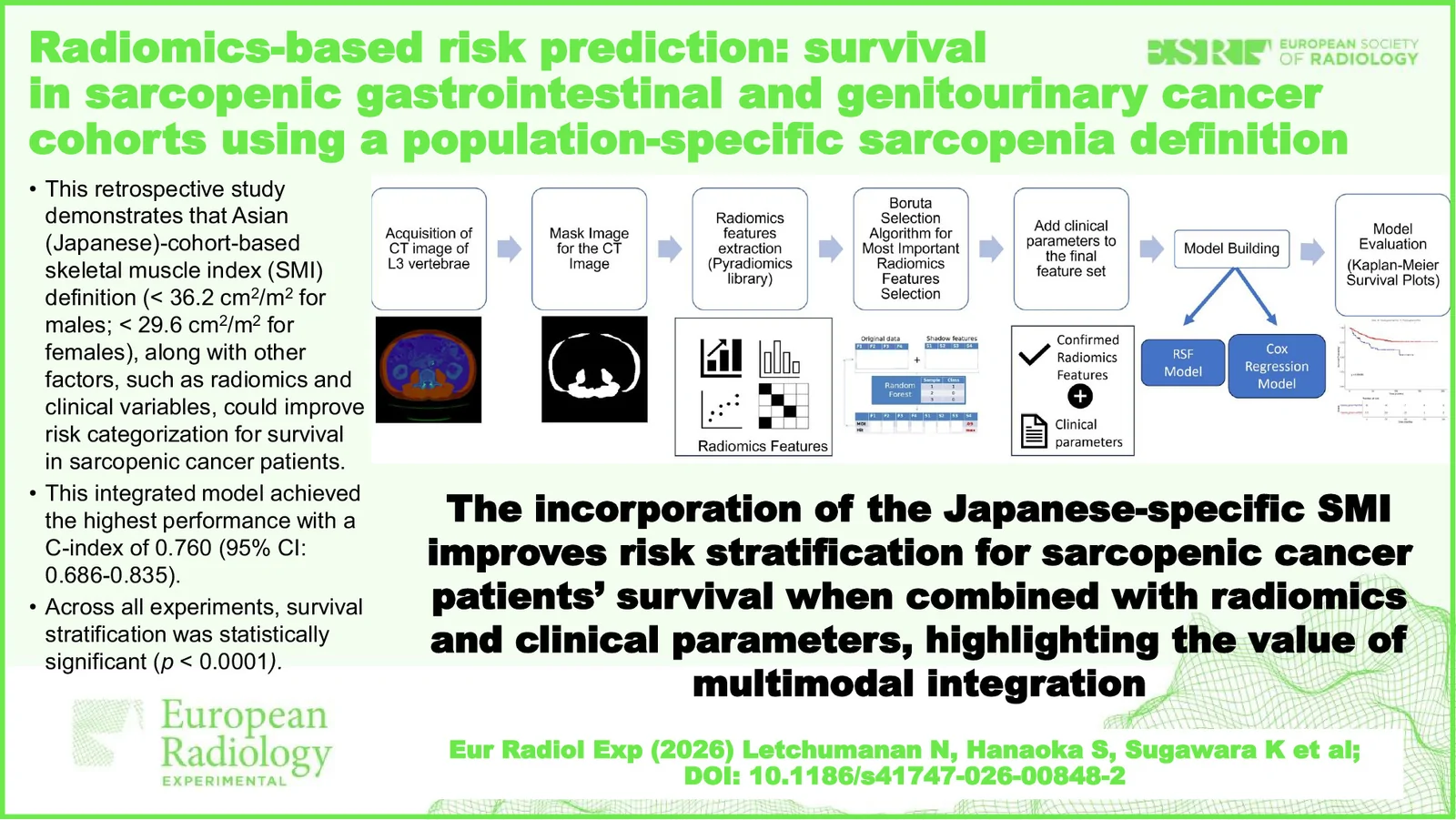

## Background

In oncology, sarcopenia is often associated with poor clinical outcomes, underscoring its relevance as a prognostic factor in cancer patients [1–3]. The European Working Group on Sarcopenia in Older People 2 (EWGSOP2) has defined sarcopenia as a progressive and widespread skeletal muscle condition associated with a greater risk of adverse outcomes [4]. A patient is considered sarcopenic when low muscle mass is accompanied by either low muscle strength or poor physical performance [5, 6].

Whole-body muscle composition is known to be correlated with the third lumbar vertebra (L3) area from computed tomography (CT) images, where it can also indicate reduced muscle mass and strength [7]. A standardized measure of muscle mass can be derived from the skeletal muscle index (SMI), $\left(\frac{{{cm}}^{2}}{{m}^{2}}\,\right)$ which is the cross-sectional area of all skeletal muscles in the L3 vertebral plane and is known as the skeletal muscle area (SMA, ${{\rm{cm}}}^{2}$) divided by the height in meters squared (${\rm{m}}^{2}$) [4].

However, there are no globally standardized SMI cutoff values for sarcopenia due to differences in ethnicity and body composition [2]. This study utilized SMI cutoff values specifically curated from a Japanese cohort [3]. This definition, called the Fujiwara et al definition throughout this study, was used to predict the survival of cancer patients in terms of overall survival (OS).

We employed the radiomic technique, which enables the extraction of quantitative features from medical images and converts them into analyzable data for clinical decision-making support [8, 9]. Many studies have explored the feasibility of using radiomic features to predict the survival outcomes of patients with cancer who develop sarcopenia [10–12].

Nevertheless, to our knowledge, the methodology used in this study is novel because of the two distinct modifications that have been made. First, we used the Boruta algorithm to select important radiomic features, unlike many other studies that solely depended on multivariate Cox regression analyses [13, 14] or the least absolute shrinkage and selection operator (LASSO) method [11]. We also incorporated a new definition of sarcopenia by Fujiwara et al on the basis of the Japanese population, which best suited our patient cohort.

The prognostic importance of incorporating Asian (Japanese)-specific SMI cutoff values with radiomic and clinical parameters remains largely unexplored. Hence, the primary aim of this study was to evaluate survival outcomes (OS) *via* the Fujiwara et al SMI cutoff thresholds. We also examined whether the radiomic features extracted from the L3 vertebral region add prognostic value and whether integrating clinical parameters, radiomic features, and Asian (Japanese)-specific SMI thresholds enhances survival risk stratification compared with standalone models (models built with a single predictor category, such as clinical parameters alone, radiomic features alone, or population-specific SMI thresholds alone).

## Materials and methods

### Study design and patient data

This single-center, retrospective study was approved by our Institutional Review Board with approval numbers 2561-(29) and 2022046NI. The need to obtain written consent was waived. This study followed the Checklist for Artificial Intelligence in Medical Imaging (CLAIM) [15] for methodological validity.

The patient data were obtained from our previous study [1] (permission to reuse from Elsevier, license number: 5683921215082). The data spanned from January 2006 to December 2018 and included 2,869 patients with gastrointestinal (GI) (gastric cancer, *n* = 1,262; esophageal cancer, *n* = 596) or genitourinary cancer (GU) (renal cell cancer, *n* = 554; urothelial cancer, *n* = 457). All patients underwent major surgery at The University of Tokyo Hospital. A summary of the surgical procedures performed on the patients is presented in Table S1. Patients with complete clinical data (age, sex, cancer type, stage, and survival outcomes) and preoperative CT images obtained within 3 months were included. Patients with overlapping surgical histories, including esophagectomy after prior gastrectomy (*n* = 22) and cystectomy after prior nephroureterectomy (*n* = 7), were excluded. The final cohort comprised 2,015 patients with gastrointestinal (GI) (gastric cancer, *n* = 917 or esophageal cancer, *n* = 465) and genitourinary cancer (GU) (renal cell cancer, *n* = 384 or urothelial cancer, *n* = 249).

### CT images acquisition and definition of SMI for sarcopenia

CT images were acquired for over 12 years *via* various CT scanners from five manufacturers, namely Toshiba/Canon models (Aquilion, Aquilion ONE, Aquilion PRIME, Aquilion Precision), GE Medical Systems models (Discovery CT570 HD, LightSpeedVCT), and scanners from Siemens, Philips, and Hitachi. The slice thickness was 5.0 mm, and the tube voltage was 120 kVp. The reconstruction kernels varied across all the scanners according to vendor-specific reconstruction filters. The DICOM (Digital Imaging and Communications in Medicine) images were de-identified *via* the Python *pydicom* library by removing patient-specific metadata from the DICOM headers.

We utilized axial L3 CT images, in which the SMA was measured *via* an internal computer-aided diagnosis (CAD) platform known as CIRCUS version 1.6.0 [16]. This platform was built for a semiautomatic segmentation task *via* deep learning algorithms. The measured SMA was used to calculate the SMI $\left(\frac{{{cm}}^{2}}{{m}^{2}}\,\right)$. Sarcopenia was defined according to the SMI cutoff values of Fujiwara et al (< 36.2 ${{\rm{cm}}}^{2}\rm/{m}^{2}$ for males and < 29.6 ${{\rm{cm}}}^{2}\rm/{m}^{2}$ for females) [3].

### Survival prediction workflow

**Fig. 1:**
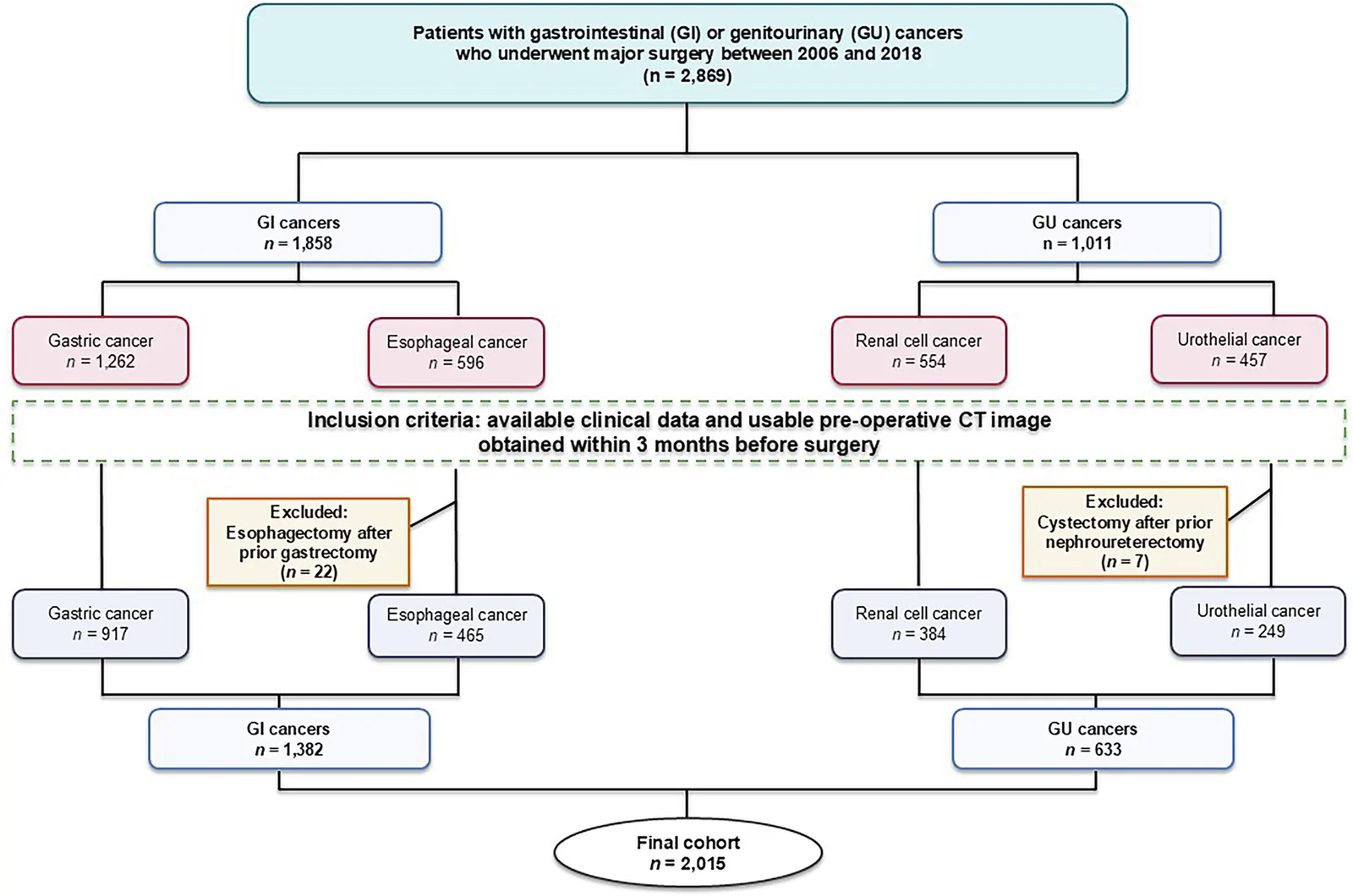
Overall framework of the study. RSF, Random forest survival

This study utilizes radiomics combined with other factors, such as clinical parameters and the definition of Fujiwara et al, to assess the survival prediction of sarcopenic patients *via* a machine learning (ML) model called random survival forest (RSF) [17] and the multivariate Cox proportional hazard model. The overall framework of the study is shown in Fig. 1.

First, the CT image at the L3 vertebral level, from which the skeletal muscle area was delineated, and its corresponding mask image were generated. The radiomic features were subsequently extracted from the original mask images and their variations *via* various filters. The Boruta selection algorithm was used for radiomic feature selection, and the chosen features were combined with clinical parameters for further analysis.

The clinical parameters included as predictive features were body mass index (BMI), skeletal muscle area (SMA), and skeletal muscle density (mSMD). We conducted four different experiments that utilized RSF [17] and a multivariate Cox proportional hazard model to predict the survival of sarcopenic patients (Fig. S1). To divide patients into high- and low-risk categories, we used median values as cutoff points.

### Radiomics

Radiomics is a quantitative, noninvasive image analysis technique used to extract reproducible features from medical images that are beyond conventional visual assessment [9, 18]. These features are often integrated with other patient information to support clinical decision-making [9].

This study used radiomics to predict the survival curves of sarcopenic patients together with clinical parameters and the Fujiwara et al definition, and compared the differences in their performance. First, segmentation of the abdominal muscle area on a single axial CT slice at the L3 vertebral level was performed. The 2-dimensional region of interest (ROI) was delineated *via* a deep learning method and then modified by two trained radiologists with 24 and 22 years of experience. A binary mask image was created for these images, as shown in Fig. S2. This ROI is anatomically useful because most trunk skeletal muscles are located cranio-caudally, and axial slices of the L3 vertebral region provide a cross-sectional view perpendicular to the muscle fibers. This allows the 2D radiomics features to depict muscle texture and intramuscular fat characteristics. This approach was chosen to comply with the widely used method of delineating the cross-sectional skeletal muscle area at the L3 level as a surrogate marker of whole-body skeletal muscle mass in CT-based sarcopenia assessment [1].

Radiomic features were extracted *via* the Python programming language (version 3.10.5) and the *PyRadiomics* library (version 3.0.1) [19]. The number of features extracted from the feature classes was 646, namely, shape-based features, first-order statistics, the gray-level co-occurrence matrix (GLCM), the gray-level run length matrix (GLRLM), the gray-level size zone matrix (GLSZM), the neighboring gray tone difference matrix (NGTDM), and the gray-level dependence matrix (GLDM). Table S2 lists the feature classes, the number of radiomic features extracted from each class, and the image domains from which the features were computed. These features were standardized by the Image Biomarker Standardization Initiative (IBSI) [20].

### Boruta selection algorithm

The Boruta selection algorithm is a random forest model-based feature selection algorithm that leverages an ensemble method [21]. It provides a numerical value for each attribute and determines the important features *via* a systematic method, as shown in Fig. S3.

We implemented the Boruta selection algorithm to select radiomic features useful for predicting the survival curves of sarcopenic patients. This was carried out *via* R (version 4.3.2) and its Boruta library (version 8.0.0). To ensure reproducibility, a fixed random seed was set before data partitioning and Boruta-based feature selection. Boruta is preferred as the feature selection method because of its ability to handle high-dimensional data and its transparency [22].

### Experiments

#### RSF-based radiomics model (Experiment 1)

In Experiment 1, the goal was to build an RSF model with radiomic features selected by the Boruta algorithm. We used the *randomForestSRC* package from the R programming language and trained the RSF model with the “confirmed features” on *train1* by building 1,000 trees, a minimum terminal node size of five, and 50 random split points. As the RSF is an ensemble method, the algorithm aggregates predictions from individual survival trees to compute survival values without applying manual tree weights.

The predicted survival values were applied to the *train2* cohort. The median model-estimated survival values in *train2* were used as a fixed cutoff point to derive the RSF High/Low strata in both *train2* and the test set. The RSF low-risk group was categorized as patients whose predicted survival values were below the median, and those whose values were equal to or above the median were categorized as the RSF high-risk group. The Kaplan‒Meier (KM) survival curves were plotted using the test cohort for the RSF-derived high *versus* low groups to compare their survival.

#### Cox proportional hazard-based model (Experiment 2)

The “confirmed features” derived from the Boruta selection method were added to the additional clinical parameters, such as BMI, SMA, and mSMD, as the final feature set. We then fitted the multivariate Cox proportional hazard model using the final feature set as covariates on *train2* to determine the hazard ratios and derive a radiomic score (Rad-Score).

The model estimated the regression coefficients (β) *via* partial likelihood maximization, producing feature-specific log-hazard ratios. For each patient, i, the Rad-Score is calculated as the linear predictor of feature values, ${x}_{{ij}}$ and their Cox regression coefficients, ${\beta }_{j}$. Hence, the Rad-Score can be defined as:

$${Rad}{\mbox{-}}{Score}_{i}={\sum }_{j=1\,}^{p}{B}_{j}{x}_{{ij}}$$

where, j is the radiomic feature index, p is the total number of selected features, ${x}_{{ij}}$ is the value of feature j for patient i and ${B}_{j}$ is the Cox regression coefficient (log-hazard ratio) for feature j.

The median Rad-Score was calculated *via* the *train2* set and used as the cutoff point to stratify patients into high- and low-risk groups. Patients were divided into rad-group high-risk groups if the Rad-Score ≥ median Rad-Score, and those with a Rad–Score < median Rad-Score were considered the low-risk group. This value was fixed and applied to the independent test, thereby preventing information leakage.

#### Fujiwara SMI definition-based model (Experiment 3)

Fujiwara et al–based SMI definition used to classify sarcopenia is defined as SMI < 36.2 ${{\rm{cm}}}^{2}\rm/{m}^{2}$ for males and SMI < 29.6 ${{\rm{cm}}}^{2}\rm/{m}^{2}$ for females [3]. Next, we stratified the Fujiwara high-risk group as sarcopenic patients, and those who are non-sarcopenic as the Fujiwara low-risk group. Survival differences between groups were directly evaluated *via* Kaplan–Meier analysis and log-rank tests.

#### Integrated model (Experiment 4)

In the final experiment, we assessed the ability of combined parameters, such as radiomic features, clinical parameters, and the Fujiwara et al definition proposed for sarcopenia, to predict the survival of sarcopenic patients. As described in the previous sections, the radiomic-clinical signature-based risk groups derived from Experiment 2 and the risk groups categorized on the basis of the Fujiwara et al definition for sarcopenia from Experiment 3 were used in the fourth experiment.

This integrated, rule-based model was composed of patients who were categorized as high-risk by both the Fujiwara-defined sarcopenia group and the radiomic risk group approach; hence, it should not be interpreted as an ensemble model. These patients were classified into the DoubleHigh-risk group, and the remaining patients were grouped together for comparison. KM curve analysis was performed for survival prediction, and the log-rank test was used to assess statistical differences.

### Experimental settings

The dataset was initially divided at the patient level into a ratio of 75:25, *n* = 1,512 for training and *n* = 503 for the testing phase. The subsets were mutually exclusive, without an overlap of patients between the two sets. Model evaluation was performed using only the held-out internal test cohort. The training cohort was further split into two equal subsets, namely *train1* and *train2*. *Train1* is solely for the radiomic features selection process *via* the Boruta algorithm (Experiment 1, 2, and 4) and for the RSF model building. Boruta is applied to *train1*, and the “confirmed features” are determined.

Additional clinical parameters, such as BMI, SMA, and mSMD, were added to the “confirmed features” to form the final feature set. We fitted the multivariate Cox proportional hazard model using the final feature set as covariates on *train2*, to determine the hazard ratios and derive the Rad-Score.

We binarized the risk groups *via* *train2*-derived median values (Rad-score), and these values were treated as fixed thresholds and subsequently applied to the independent test set. The outcomes of Experiments 3 and 4 were predefined risk-group stratifications and were evaluated as censored time-to-event outcomes. Model performance was evaluated *via* survival-specific metrics, such as KM survival curves and log-rank tests, with a *p*-value < 0.05 considered statistically significant. The concordance index (C-index) with 95% confidence intervals (CIs) was computed to quantify the predictive accuracy of the models. Pairwise comparisons of C-index values were computed whenever necessary *via* the *compareC* package from R. To ensure the robustness of the integrated model, we performed an exploratory, repeated-seed sensitivity test. The internal *train1/train2* split and the model fitting were repeated across 10 random seeds while keeping the held-out internal test cohort fixed. Model performance was evaluated by the median C-index and interquartile range (IQR).

## Results

**Fig. 2:**
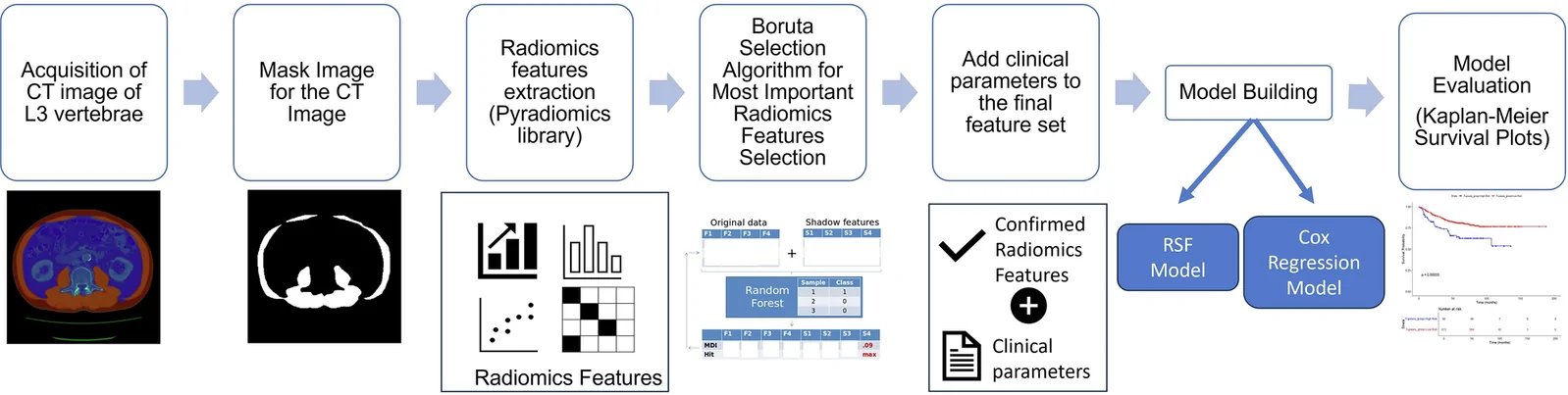
Patient selection process. Adapted from our previous publication with modifications [26]. CT, Computed tomography

The final study cohort comprised 2,015 patients. Figure 2 shows the flowchart of the patient selection process. Most patients were in the ‘Normal’ BMI category (18.5–24.9 ${\rm{kg}}/{m}^{2}$), with a median BMI of 22.4 ${\rm{kg}}/{m}^{2}$(IQR: 20.2–24.5). According to the Fujiwara et al definition, 358 patients (17.8%) were categorized as sarcopenic, and 1,657 patients (82.23%) were non-sarcopenic. During the follow-up period, 303 patients (15.0%) died of cancer, and 147 deaths were attributed to other causes. The median follow-up period was 61 months (IQR: 34–79). The details of the patient cohort are shown in Table 1 and in a previous study [1].

**Table 1:** Patient demographics of the full cohort and analytical subsets

Variables	Full cohort (*n* = 2,015)	Training set (*n* = 1,512)	Testing set (*n* = 503)
Age, median years (IQR)	67 (59–74)	67 (59–74)	68 (60–74)
Male, ${{{\rm{n}}}}$ (%)	1,494 (74.1%)	1,125 (74.4%)	369 (73.4%)
Female, ${{{\rm{n}}}}$ (%)	521 (25.9%)	387 (25.6%)	134 (26.6%)
BMI, ${{{\rm{kg}}}}/{{{{\rm{m}}}}}^{{{{2}}}}$, median (IQR)^†^	22.4 (20.2–24.5)	22.3 (20.1–24.5)	22.5 (20.3–24.6)
SMA,$\,{{{{\rm{cm}}}}}^{{{{\rm{2}}}}}$, median (IQR)	108.5 (88.8–126.0)	108.8 (88.7–126.4)	108.2 (89.0–125.6)
SMI, ${{{{\rm{cm}}}}}^{{{{2}}}}/{{{{\rm{m}}}}}^{{{{2}}}}$, median (IQR)	40.3 (35.1–45.4)	40.3 (35.1–45.4)	40.3 (35.4–45.5)
mSMD, HU, median (IQR)	44.3 (38.4–49.9)	44.5 (38.4–50.0)	43.8 (38.8–49.6)
SMI (Fujiwara et al), ${{{{\rm{cm}}}}}^{{{{2}}}}/{{{{\rm{m}}}}}^{{{{2}}}}$, ${{{\rm{n}}}}$ (%)^††^	358 (17.8%)	265 (17.5%)	93 (18.5%)

*BMI* Body mass index, *SMA* Skeletal muscle area, *SMI* Skeletal muscle index, *mSMD* Mean skeletal muscle density

^†^ Normal BMI range: 18.5–24.9 ${{{\rm{kg}}}}/{{{{\rm{m}}}}}^{{{{2}}}}.\,$

^††^ Sarcopenia, according to Fujiwara et al was defined as sex-specific SMI cutoff values: < 36.2 ${{{{\rm{cm}}}}}^{{{{2}}}}/{{{{\rm{m}}}}}^{{{{2}}}}$ for males and < 29.6 ${{{{\rm{cm}}}}}^{{{{2}}}}/{{{{\rm{m}}}}}^{{{{2}}}}$ for females

### Feature selection

Among the 646 radiomics features, the Boruta algorithm identified only four features as important. The selected features and their feature classes are listed in Table 2.

**Table 2:** Boruta selected radiomic features and their classes

Feature name	Feature class	Calculated for:
10th percentile	First-order	Log-sigma-3.0 mm Log-sigma-4.0 mm
High gray-level zone emphasis (HGLZE)	Gray-level size zone matrix (GLSZM)	Original
Gray-level non-uniformity (GLN)	Gray-level dependence matrix (GLDM)	Wavelet-L

The Rad-Score formula derived from the Boruta-selected radiomic features, clinical parameters, and Cox model coefficients is as follows:

$${Rad}{\mbox{-}}{Score}= 	 \,{HGLZE}^\ast \left(0.0023\right)\\ 	 +10{th}\,{Percentile}\,{logsigma}\,3{mm}^\ast \left(0.2476\right)\\ 	 +10{th}\,{Percentile}\,{logsigma}\,4{mm}^\ast \left({\mbox{-}}0.1978\right)\\ 	 +{GLN}^\ast \left({\mbox{-}}0.0001\right)+{BMI}^\ast \left({\mbox{-}}0.1630\right)\\ 	 +{SMA}^\ast \left(8.6116e-05\right)+{mSMD}^\ast ({\mbox{-}}0.0385)$$

The median cutoff points for the Rad-Score and RSF survival month predictions were -4.79 and 11 months, respectively. These thresholds were fixed and applied to the test set.

### Kaplan‒Meier survival curves

**Fig. 3:**
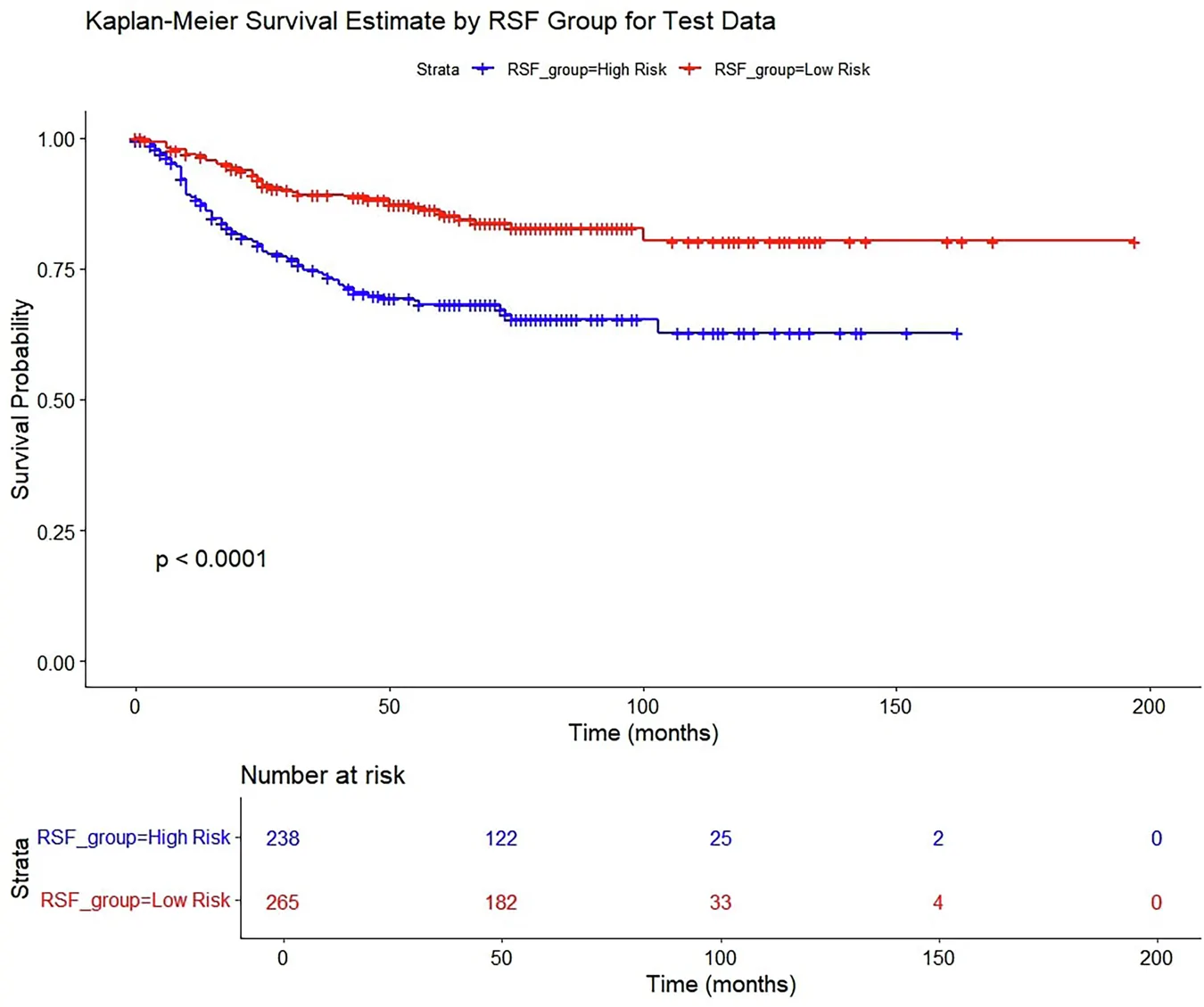
KM survival curve for radiomics only (Experiment 1). The risk table shows the number of patients classified in the high- and low-risk groups, respectively. The curves show clear separation between the high- and low-risk groups

**Fig. 4:**
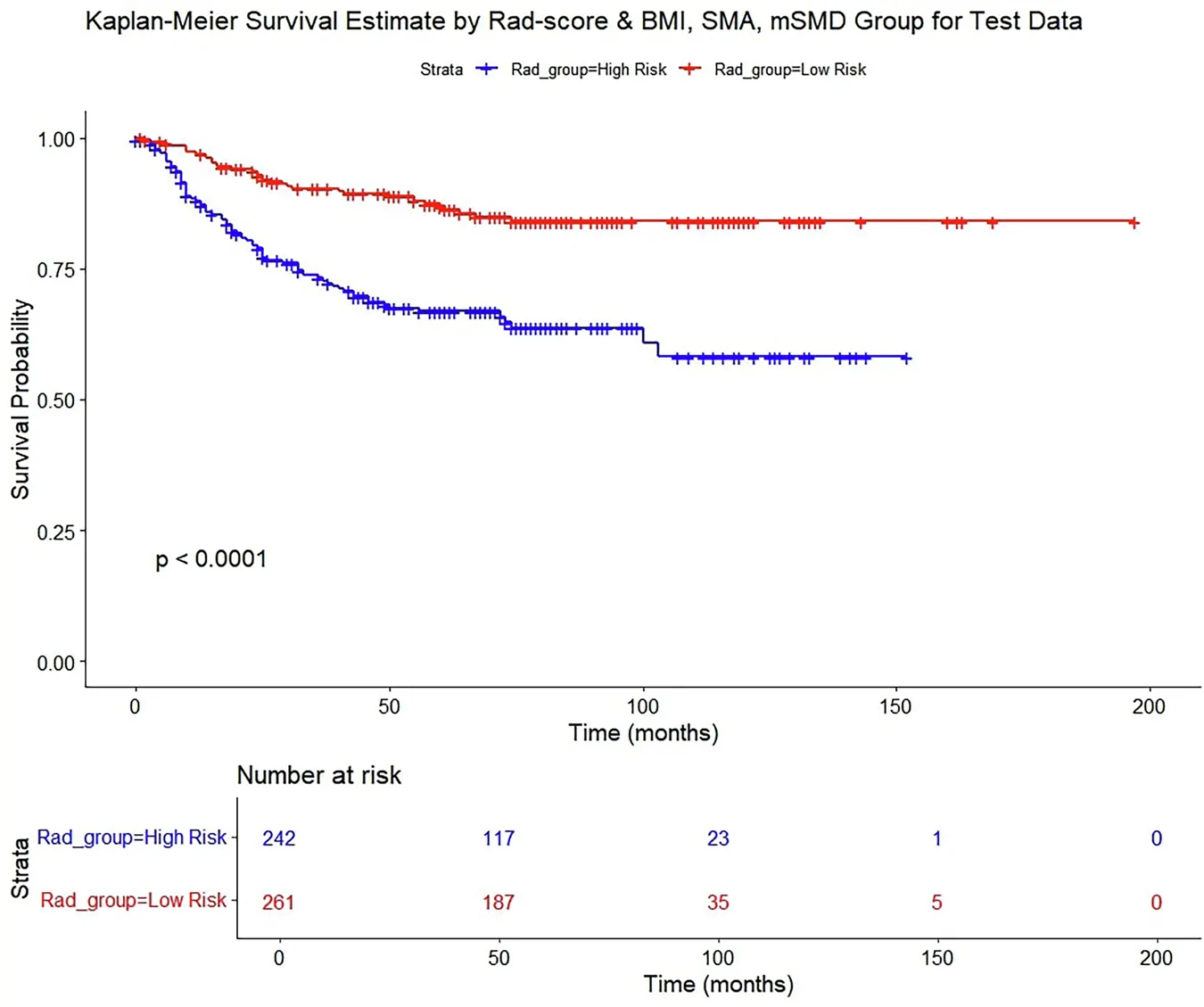
KM survival curve for radiomics and clinical parameters (Experiment 2) shows a visible separation between the high- and low-risk groups

**Fig. 5:**
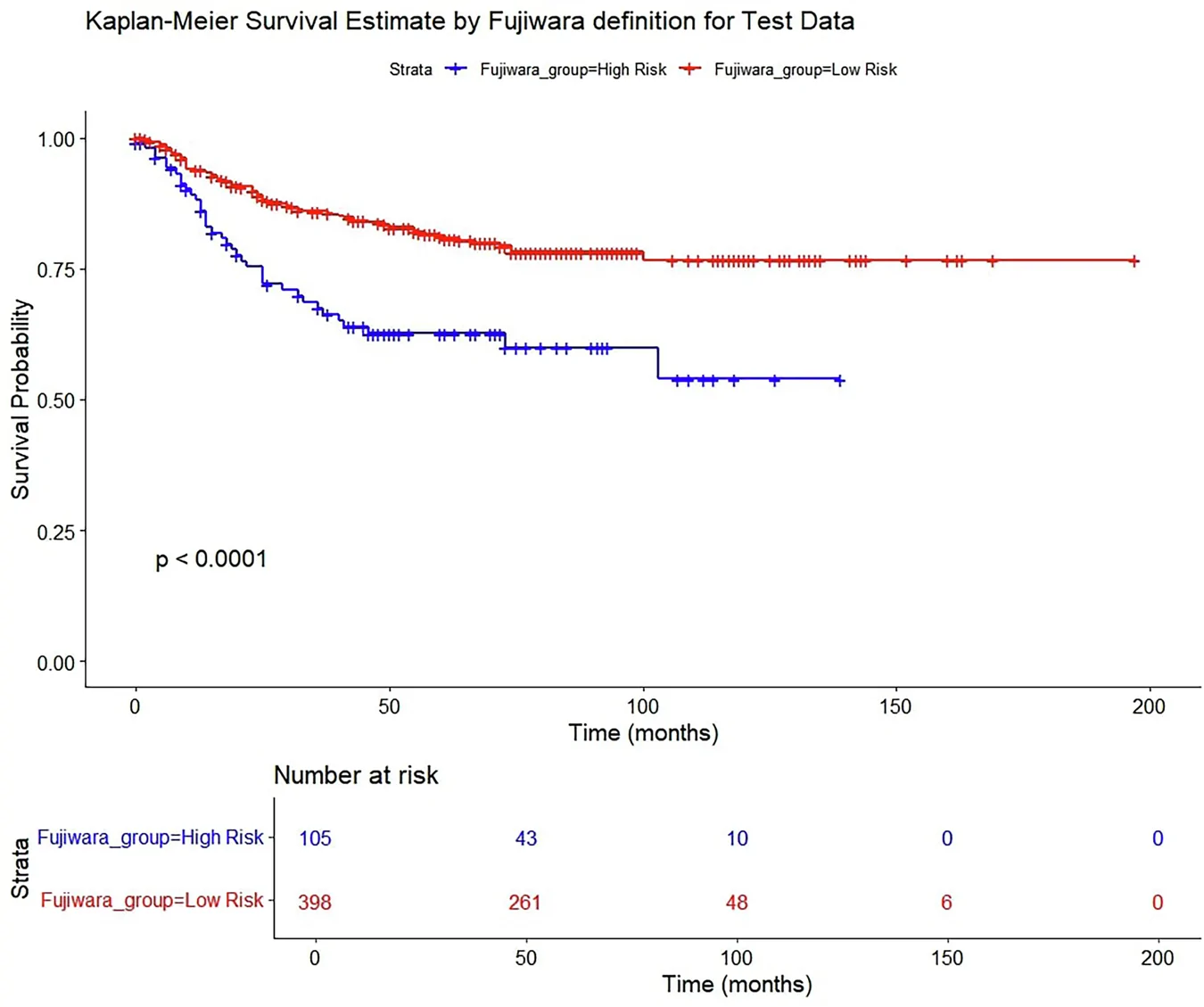
KM survival curve for Fujiwara et al definition (Experiment 3). Distinct divergence between the KM curves for high- and low-risk groups can be observed

**Fig. 6:**
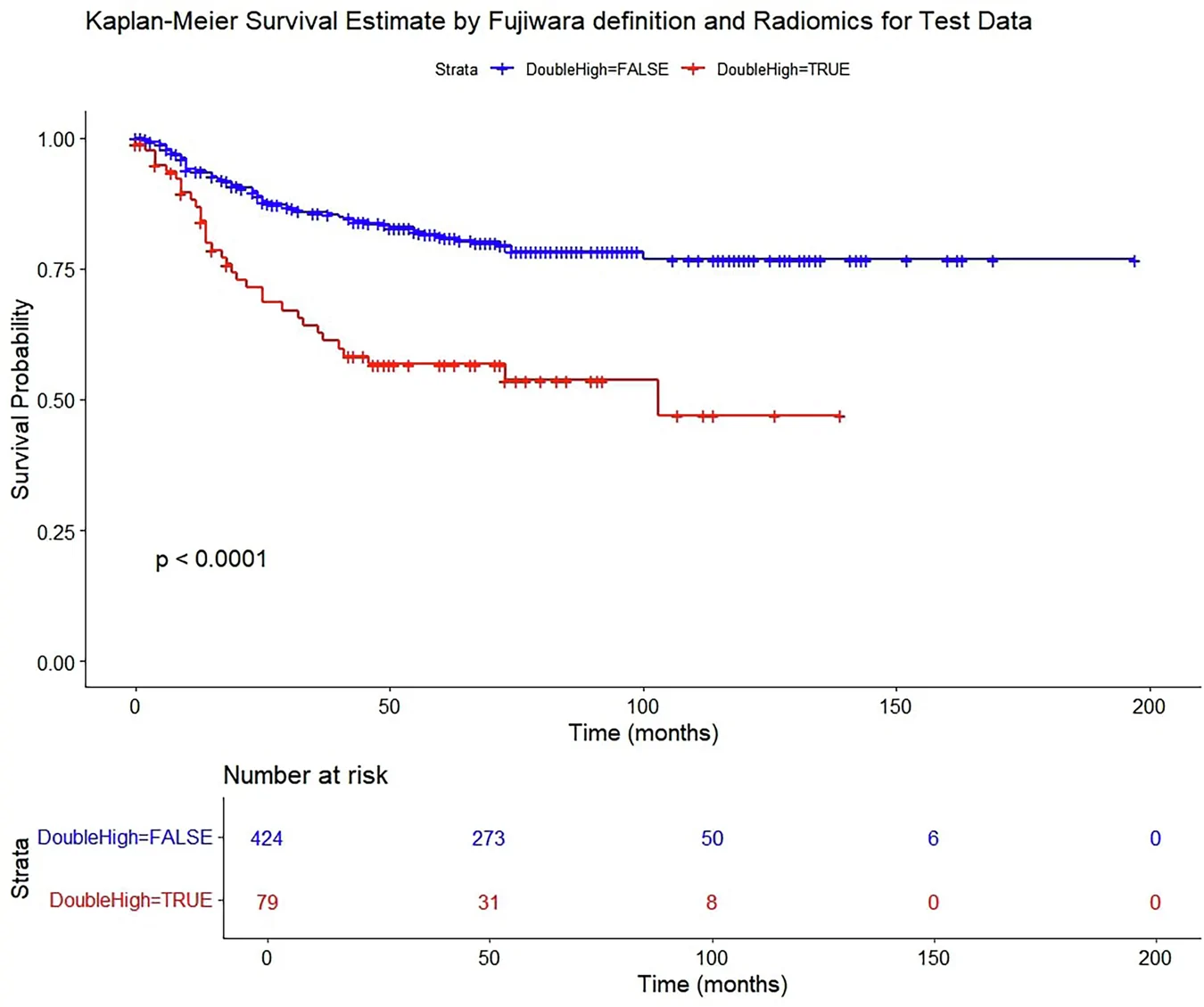
KM survival curve for Fujiwara et al definition and radiomics (Experiment 4). DoubleHigh: patients with high risk by both Fujiwara et al criteria and radiomics score. Patients with a lower risk for both the Fujiwara group and the radiomics group survive longer than patients with a higher risk for both groups

Figures 3–6 illustrate the KM plots with corresponding risk tables, which highlight the number of patients classified into high- and low-risk groups at each time point. A log-rank test and C-Index with 95% CI were computed, and the results are tabulated in Table 3. All the metrics reported refer to the independent test set, with cut points derived on *train2*. The RSF-based radiomic model stratified patients into high- and low-risk groups with visibly separated KM curves, as shown in Fig. 3. The statistical analyses revealed that the model achieved a C-index of 0.728 (95% CI: 0.648–0.807; *p* < 0.0001). The incorporation of clinical parameters (BMI, SMA, and mSMD) into the radiomic model resulted in a numerically greater C-index of 0.750 (Fig. 4); however, this difference was not statistically significant compared with that of the RSF-based radiomic model (*compareC*, *p* = 0.380). The Fujiwara model (Fig. 5) achieved a numerically lower C-index of 0.706 (95% CI: 0.621–0.791), with the separation between the curves being statistically significant (*p* < 0.0001). The integrated approach with the radiomic and clinical parameters combined with the Fujiwara et al definition achieved the numerically highest C-index of 0.760 (95% CI: 0.686–0.835), demonstrating significant discriminative ability (*p* < 0.001) (Fig. 6). Pairwise comparison *via* *compareC* revealed that the integrated model had a significantly higher C-index than the Fujiwara-only model did (*p* = 0.016). The exploratory, repeated-seed sensitivity analysis for the high-performing, integrated model revealed stable performance, with a median C-index of 0.758 (IQR: 0.750–0.764), consistent with the primary test-cohort result.

**Table 3:** Survival performance of the models

Model	*p*-value	C-index (95% CI)
Radiomics only	< 0.0001	0.728 (0.648–0.807)
Radiomics + clinical parameters		0.750 (0.673–0.828)
Fujiwara et al definition for sarcopenia		0.706 (0.621–0.791)
Radiomics + clinical parameters + Fujiwara et al definition for sarcopenia (integrated model)		0.760 (0.686–0.835)

## Discussion

The best overall performance was achieved *via* the integrated model combined with radiomic-clinical risk stratification and the Fujiwara et al SMI definition. The model achieved the highest C-index value of 0.760 (95% CI: 0.686–0.835). A clear separation of the KM curves (*p* < 0.0001) demonstrated that the patients categorized as high-risk by both the radiomic-clinical model and the Fujiwara et al model had a lower survival probability. This model also achieved a significantly greater C-index than did the Fujiwara-only model (*compareC*, *p* = 0.016), indicating that muscle characteristics derived *via* radiomics and population-specific SMI thresholds may provide complementary prognostic information.

This may be because the model was presented with Boruta-selected radiomic features during training; hence, it could have learned the associations between the features and CT images, which cumulatively led to better performance [14]. The radiomic features derived from the CT images of L3 skeletal muscle areas with the Fujiwara et al definition for SMI were able to predict sarcopenic patients’ survival, especially when combined with clinical parameters. This finding supports the prognostic importance of utilizing skeletal muscle segmentation effectively at the L3 vertebral level [4, 23].

Population-specific clinical cutoff values, such as the SMI, have also been reported to contribute to survival prediction in several previous studies [13, 23–26]. For example, a study from Canada, which integrated sarcopenic status, radiomics, and clinical parameters, concluded that the combination of these covariates may enhance survival prediction in patients with gastroesophageal cancer [13]. They used SMI values from [23] derived from patients in the United States. Similarly, a Brazilian study [24] highlighted that population-based cutoff values are important for examining the CT scans of cancer patients because body composition usually differs across countries. Another study from China, which investigated the survival complications of gastric cancer patients *via* radiomics and sarcopenia definitions, also emphasized the importance of using population-specific SMI values (40.8$\,{{\rm{cm}}}^{2}\rm/{m}^{2}$ for men and 34.9$\,{{\rm{cm}}}^{2}\rm/{m}^{2}$ for women) tailored for the Chinese population [25]. Our findings are consistent with those of previous studies, reinforcing the prognostic relevance of the use of SMI cutoff values reported by Fujiwara et al in Japanese patients.

Furthermore, radiomics features handpicked by Boruta, such as the GLN and HGLZE, play a vital role in this prognostic task. The GLN measures the variability of gray levels within the muscle [20], whereas the HGLZE captures the emphasis on large, high-intensity homogeneous zones [20]. Both features, together with low-intensity percentile descriptors, capture heterogeneity within muscle signals that standard measures may miss.

The model that used Fujiwara et al’s definition alone presented the lowest C-index numerically: 0.700 (95% CI: 0.621–0.791). This may be because SMI thresholds alone lack the ability to capture sufficient information to make accurate predictions, compared with the enhanced ability of CT-derived radiomic features.

From a clinical perspective, improved survival risk stratification *via* preoperative CT scans and clinical parameters may benefit patients who require closer monitoring or individualized supportive care [13]. However, at the current stage of this study, the models should be regarded as research tools, as they require extensive clinical validation *via* external datasets. Nevertheless, our Boruta-derived feature selection strategy provides a reproducible basis for external validation and further refinement in independent cohorts.

This study has several limitations. First, the Boruta algorithm used in this study for radiomic feature selection may be affected by the “maxRuns” stopping criterion, which stops the random forest runs, leaving some important features as “Tentative Attributes” [21]. Second, we employed traditional radiomics features directly from *PyRadiomics*, which have explainability and reproducibility. However, researchers have reported that deep learning radiomics (DLR) can perform better and has a strong ability to predict patient prognosis, as reported in these studies [12, 27, 28]. Moreover, this study was conducted solely on a Japanese population, limiting its potential for generalizability in its current phase. External validation was not performed because of data scarcity, which remains a prospect for future studies.

Next, the number of clinical variables included in this study was limited, and other contributors to sarcopenia, such as genetics, lifestyle factors, and the presence of other diseases, were not considered. As the evaluation of the models focused on time-to-event risk, a detailed case-specific failure analysis was not performed. Future studies should emphasize discordant scenarios, such as low-risk patients with early death and high-risk patients with prolonged survival.

Another limitation is that the ROI was limited to a single axial CT slice at the L3 vertebral level. Thus, the extracted radiomic features may not capture the characteristics of skeletal muscle as comprehensively as 3-dimensional volumetric radiomic features do. Furthermore, CT scans were retrieved from various scanners over a 12-year study period. Although this may influence the radiomic features, it also reduces the reliance on a single scanner and may better reflect real-world clinical practice.

## Conclusion

In conclusion, this study presents a new idea for implementing the recently published Fujiwara et al definition for SMI cutoff values along with radiomics features and clinical values to strengthen the risk stratification for sarcopenic cancer patients (C-index: 0.760; 95% CI: 0.686–0.835). These results highlight the importance of a multimodal approach for better survival risk stratification.

## Supplementary information

**Additional file 1: Table S1:** Surgical Procedures Done on GI or GU Cancer Patients. **Table S2:** Radiomics feature classes and their corresponding features. **Figure S1:** Details on the experiments conducted in this study. **Figure S2:** a) The Original CT Image of L3 vertebrae, where the muscle area is delineated by a trained radiologist. (b) The binary mask of the CT image. Figure S3: Boruta Selection Process, adapted from [21].

## Data Availability

The dataset and code are not publicly available to preserve patient confidentiality.

## Abbreviations

BMI: Body mass index
CI: Confidence intervals
GI: Gastrointestinal
GLN: Gray-level non-uniformity
GU: Genitourinary
HGLZE: High gray-level zone emphasis
IQR: Interquartile range
KM: Kaplan–Meier
mSMD: Mean skeletal muscle density
RSF: Random survival forest
SMA: Skeletal muscle area
