# Supplementary material for: Radiomics-based risk prediction: survival in sarcopenic gastrointestinal and genitourinary cancer cohorts using a population-specific sarcopenia definition
Source: Eur Radiol Exp. 2026 Nov 13;10:178. doi: 10.1186/s41747-026-00848-2 (PMC13768411; doi:10.1186/s41747-026-00848-2)
Supplement: Supplementary file 1 [file 41747_2026_848_MOESM1_ESM.pdf]

Radiomics-based Risk Prediction: Survival in Sarcopenic Gastrointestinal and Genitourinary Cancer Cohorts Using a Population-Specific Sarcopenia Definition

ELECTRONIC SUPPLEMENTARY MATERIAL

Table S1: Surgical Procedures Done on GI or GU Cancer Patients

Cancer	Cancer Type	Surgeries Conducted
Gastric Cancer	GI	Total Gastrectomy<br>Distal Gastrectomy<br>Proximal Gastrectomy
Esophageal Cancer	GI	Esophagectomy
Renal Cell Cancer	GU	Radical Nephrectomy<br>Partial Nephrectomy
Urothelial Cancer	GU	Radical Cystectomy and/or<br>Radical Nephroureterectomy

Table S2: Radiomics feature classes and their corresponding features.

Features Class	Number of Features	Calculated for:
Shape-based features	14	Original image mask
First-order features	144	Original image mask<br>Log-sigma-2.0mm<br>Log-sigma-3.0mm<br>Log-sigma-4.0mm<br>Log-sigma-5.0mm<br>Wavelet-L<br>Wavelet-H
GLCM	176	
GLRLM	120	
GLSZM	128	
GLDM	112	
NGTDM	40	
Total	646	

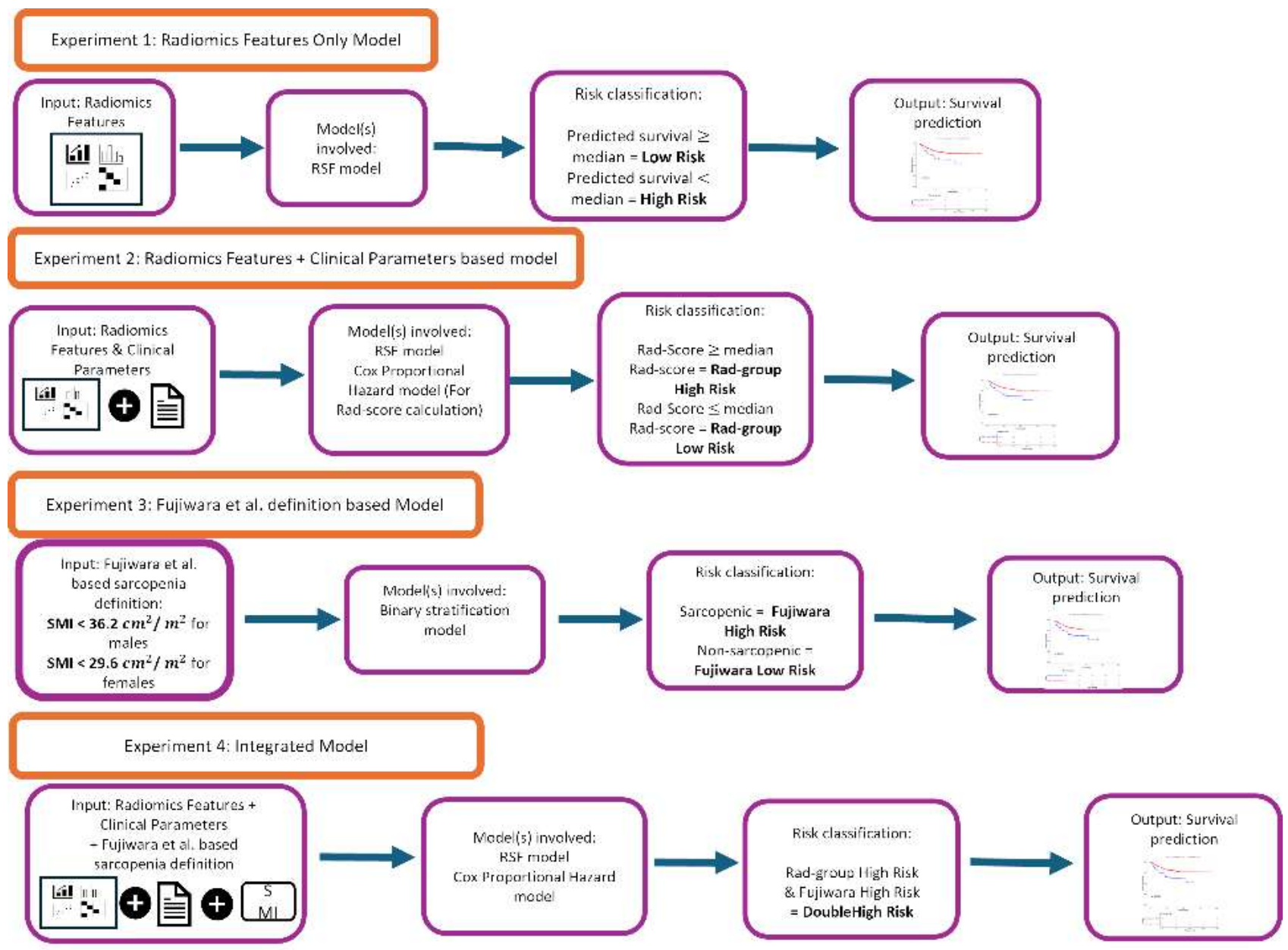

Figure S1: Details on the experiments conducted in this study.

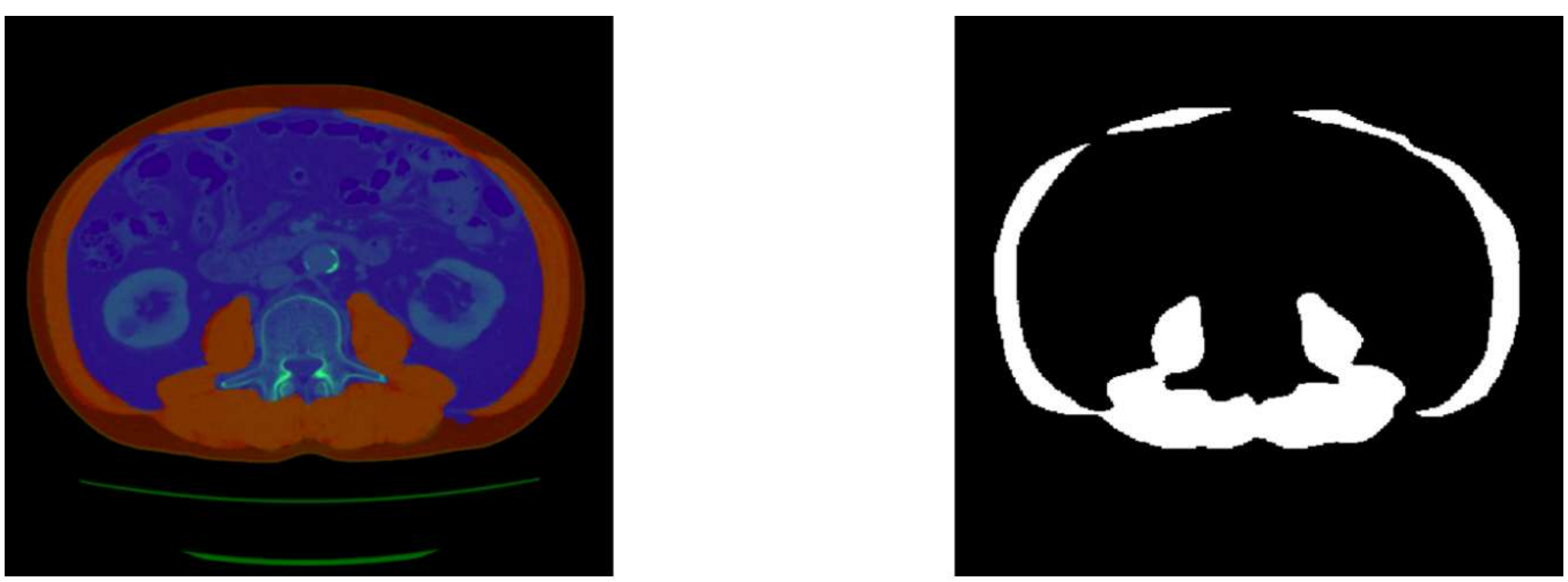

Figure S2: a) The Original CT Image of L3 vertebrae, where the muscle area is delineated by a trained radiologist. (b) The binary mask of the CT image.

Boruta Selection Process:

1. Create shadow attributes by copying the variables to expand the information available.
2. Shuffle the features so there is no correlation with the response.
3. Perform random forest classification and get the Z scores.
4. Determine the maximum Z score among shadow attributes (MZSA), then mark every attribute that scores higher than this maximum as a hit.
5. Two-sided test of equality with MZSA for features with undetermined importance
6. Selection:
    a. Attributes with less importance than MZSA: classified as "unimportant" features and are deleted from the system
    b. Attributes with more importance than MZSA: classified as "important" features
7. Shadow features are removed.
8. Repeat the selection process until all attributes have an importance value or until the limit of the random forest runs set previously in the algorithm.

Figure S3: Boruta Selection Process, adapted from [21]
